# What explains the link between romantic conflict with gambling problems? Testing a serial mediational model

**DOI:** 10.3389/fpsyg.2023.1018098

**Published:** 2023-07-12

**Authors:** Amanda E. F. Hagen, Raquel Nogueira-Arjona, Simon B. Sherry, Lindsey M. Rodriguez, Igor Yakovenko, Sherry H. Stewart

**Affiliations:** ^1^Department of Psychology & Neuroscience, Dalhousie University, Halifax, NS, Canada; ^2^School of Psychology, University of Sussex, Falmer, United Kingdom; ^3^Department of Psychology, University of South Florida St. Petersburg Campus, Tampa, FL, United States; ^4^Department of Psychiatry, Dalhousie University, Halifax, NS, Canada

**Keywords:** gambling, problem gambling, romantic conflict, coping motives, negative affect

## Abstract

**Introduction:**

While individuals have many motives to gamble, one particularly risky motive for gambling is to cope with negative affect. Conflict with one’s romantic partner is a strong predictor of negative affect, which may elicit coping motives for gambling and, in turn, gambling-related problems. Support for this mediational model was demonstrated in relation to drinking-related problems. We extended this model to gambling.

**Method:**

Using a cross-sectional design, we examined links between romantic conflict (Partner-Specific Rejecting Behaviors Scale), negative affect (Depression, Anxiety, and Stress Scales-21), coping gambling motives (Gambling Motives Questionnaire, coping subscale), and gambling-related problems [Problem Gambling Severity Index (PGSI)] in 206 regular gamblers (64% men; mean age = 44.7 years; mean PGSI = 8.7) who were in a romantic relationship and recruited through Qualtrics Panels in July 2021.

**Results:**

Results supported our hypothesis that the model would explain a significant amount of variance in gambling-related problems, *β* = 0.35, 95% CI [0.24, 0.47], and that the association between romantic conflict and gambling-related problems would be sequentially mediated through negative affect and coping gambling motives, *β* = 0.07, 95% CI [0.03, 0.11], and also showed a strong single mediation pathway through negative affect alone, *β* = 0.24, 95% CI [0.16, 0.35].

**Discussion:**

Negative affect and coping gambling motives partially explain the link between romantic conflict and gambling-related problems. Interventions should target both negative affect and coping gambling motives in response to romantic conflict to reduce gambling-related problems in partnered gamblers.

## 1. Introduction

Problem gambling refers to difficulties in one or more major domains of life (i.e., financial, interpersonal, academic, and/or occupational) and associated distress as the result of gambling behavior. Problem gambling includes, at the extreme level of severity, gambling disorder (Eby et al., 2016), a diagnosis included in the *Diagnostic and Statistical Manual of Mental Disorders, Fifth Edition* (*DSM-5*) as an addictive disorder (American Psychiatric Association [APA], 2013). While gambling behavior is common (66–80%; Williams et al., 2021), only a subset of gamblers go on to develop problem gambling: estimated rates of past-year problem gambling vary from 0.6 to 5.0% and show considerable stability across time, despite overall population reductions in gambling frequency (Welte et al., 2015; Williams et al., 2021).

According to a large-scale epidemiological study by Kessler et al. (2008), most problem gamblers (96%) report comorbidity with another *DSM-5* diagnostic category, most commonly substance use (76%), anxiety (60%), and/or mood (56%) disorders. Additional evidence supports the association between gambling disorders with trauma, stress, and related disorders (Moore and Grubbs, 2021). For specific comorbid disorders, following alcohol and nicotine use disorders, major depressive disorder is the next most commonly co-occurring disorder (39%) reported amongst problem gamblers, with a significantly greater than chance occurrence (15%) of posttraumatic stress disorder (PTSD) as well (Kessler et al., 2008). Among the sub-population with both depression and problem gambling, 74% report an earlier onset of major depression, followed by onset of problem gambling, which is similar (82%) for those with both an anxiety disorder and problem gambling, while about half of those with PTSD report the PTSD preceding the onset of problem gambling (Kessler et al., 2008). Emotional disorders, such as depression, can also be a consequence of problem gambling (Dussault et al., 2011). Cross-lagged analyses support the suggestion of depressive and anxiety disorders as an etiological risk factor and possible maintenance factor for problem gambling (Kessler et al., 2008), although this finding has not always been consistent (Chinneck et al., 2016). Of note, while reviews consistently find a higher prevalence of gambling problems in men compared to women (Hing et al., 2016), findings are mixed on significant gender differences in comorbidity rates among problem gamblers, wherein some report higher comorbidity rates in women and others report no gender differences in problem gambler comorbidity (El-Guebaly et al., 2006; Desai and Potenza, 2008; Yakovenko and Hodgins, 2018).

Romantic relationships are one of the most influential interpersonal relationships in adulthood (Whisman et al., 2000; Umberson et al., 2010) and romantic relationship satisfaction is a robust predictor of overall life satisfaction (Roberson et al., 2018). Although romantic conflict is inevitable and normative, both conflict quantity and a negative or rejecting conflict style (i.e., interruption, contempt, rejection, condemnation, communication avoidance) are associated with decreased relationship satisfaction (Cramer, 2000; Murray et al., 2003; Oetzel and Ting-Toomey, 2006). Additionally, conflict quantity and style are related to a host of clinically relevant variables, such as such as depressive affect, anxious affect, stress, suicide ideation, and poor sleep quality (Mackinnon et al., 2012; El-Sheikh et al., 2013; Aloia and Solomon, 2015; Till et al., 2017).

While problem gambling is defined by negative consequences of the gambling experienced by the gambler, evidence attests the adverse consequences of excessive gambling are also experienced by those in close relationships with the individual engaging in problem gambling (Kalischuk et al., 2006). Indeed, romantic partners of problem gamblers report more romantic conflict, lower relationship satisfaction, and more distress compared to partners of non-problem gamblers (Ponti et al., 2021). Overall, compared to base rates, partnerships with a problem gambler involve more romantic conflict, including a significantly increased risk of intimate partner violence (an extreme form of relationship conflict; Dowling et al., 2016).

Mixed-methods and cross-sectional quantitative data suggest a bidirectional relationship between problem gambling and romantic conflict, i.e., both problem gambling leading to conflict and conflict leading to problem gambling (Afifi et al., 2010; Suomi et al., 2019). More research has been conducted on the former: ways in which problem gambling leads to or provokes romantic conflict. For instance, cross-lagged analyses of longitudinal data provide strong evidence for problem gambling as a predictor of subsequent decreases in relationship functioning (Cowlishaw et al., 2016). Furthermore, the mechanisms underlying the problem gambling to conflict path are widely discussed and largely intuitive (e.g., the stress associated with gambling problems may be an antecedent to conflict behaviors; or adverse financial consequences resulting from problem gambling create sources of conflict for couples; Suomi et al., 2019). On the other hand, much less is known about ways in which romantic conflict may precede or exacerbate gambling problems.

Due to high rates of comorbidity with anxiety, trauma and stress-related (e.g., PTSD), and mood disorders in samples of problem gamblers (El-Guebaly et al., 2006; Kessler et al., 2008; Chou and Afifi, 2011; Moore and Grubbs, 2021), one possible link between romantic conflict and problem gambling may be negative affect. Evidence suggests controlling for mental disorders partially attenuated the link between intimate partner violence and problem gambling, suggesting symptoms of mental disorders like depressive, anxiety, and trauma/stress disorders may partially mediate the link between intimate partner violence and problem gambling (Afifi et al., 2010). Since mood deterioration follows conflict (Choi and Marks, 2008; Prager et al., 2015) and precedes problem gambling (Coman et al., 1997; Morasco et al., 2007; Gomes and Pascual-Leone, 2014; Richard et al., 2020), it is possible that negative affect may provide an important link in explaining romantic conflict’s association with problem gambling (i.e., conflict leading to gambling problems, mediated by negative affect).

Another possible explanation for the link from romantic conflict to problem gambling is via coping gambling motives. As with other addictive behaviors (Grant et al., 2007), individuals may learn to expect certain reinforcing outcomes from gambling which may motivate future gambling behavior (Stewart and Zack, 2008). A variety of distinct motives appear to underly gambling behavior including social, enhancement, coping (Stewart and Zack, 2008), and financial motives (Schellenberg et al., 2016). This four-factor motivational model overall accounts for 31% of the variance in gambling frequency and 64% of the variance in problem gambling severity (Schellenberg et al., 2016). While enhancement motives (gambling motivated by the desire for excitement) predict gambling frequency, coping motives (gambling motivated by the desire to alleviate unpleasant internal states) uniquely predict gambling problems (Stewart and Zack, 2008; Wardell et al., 2015). Indeed, coping gambling motives are considered a primary pathway to problem gambling (Blaszczynski and Nower, 2006). Motives are believed to be a strong predictor of addictive behaviors because of their proximal temporal relationship to the behavior, as opposed to more temporally distal predictors such as personality or outcome expectancies (Cooper, 1994). Given the central role of gambling to cope with negative affect in problem gambling, coping gambling motives may be another potential mediator to explain the link between romantic conflict (which tends to produce negative affect) and problem gambling.

The link between conflict and addictive behavior via coping motives has been demonstrated in the alcohol literature, where coping drinking motives were shown to mediate the association between dyadic conflict and alcohol-related problems, both cross-sectionally and longitudinally (Lambe et al., 2015). However, the prior study did not examine gambling problems, and was conducted in a young (M_age_ = 22.13 years old, *SD* = 5.67) and age-restricted sample which limits generalizability to other stages of adulthood. Moreover, this 2015 study did not directly test a pathway from conflict to coping motives via negative affect, although motivational and learning theory would predict it in the model (i.e., that romantic conflict triggers negative affect in the gambler which motivates gambling to cope with that negative affect). Indeed, a serial mediational model (see Chinneck et al., 2018) from conflict to addictive behavior, via negative affect, and in turn coping motives, remains to be tested for any form of addictive behavior. It has yet to be determined if this effect with alcohol problems will generalize to gambling problems.

Keough et al. (2018) probed a chained mediational pathway from interpersonal difficulties to problem gambling, using attachment problems as the measure of interpersonal difficulties. They found that insecure and anxious attachment were both linked to problem gambling via depressive symptoms and, in turn, coping motives. This suggests the possibility that a similar chained mediation model might help explain the link of another form of interpersonal difficulty (i.e., romantic conflict) with problem gambling. There are several differences between this study and the present study: for instance, the authors used attachment style as a predictor and a global measure of depressive symptoms (i.e., that included depressive affect as well as other symptoms of depression such as insomnia, weight changes, etc.) as the first mediator, while the current study examined conflict behavior and negative affect (depressive affect, anxious affect, and stress), respectively. Attachment style and depressive symptoms are both quite stable across time, relative to interpersonal behaviors and negative affect, which both have an important state-like component (McCormick et al., 2017), as well as more temporally proximal predictors of subsequent coping-motived gambling.

The present study sought to expand our understanding of coping gambling motives in response to romantic conflict. This would both extend this body of knowledge from the alcohol literature (i.e., Lambe et al., 2015) to the gambling literature and additionally directly test the mediating role of negative affect in a sample of adult partnered gamblers. Specifically, we examined if romantic conflict was positively associated with gambling problems, and, whether negative affect and coping motives sequentially mediated this relationship. Each individual link in this chain has been tested and supported by the literature; however, the current study is the first to link all components together in a single model. It was hypothesized that the total indirect effects would explain a significant amount of the variance in reported gambling problems, in which more severe gambling problems associated with higher rates of romantic conflict would be partly explained by the serial pathway from more severe negative affect and endorsed coping motives. Additionally, specific indirect effects (each single mediation and the chained mediation pathways) were probed to examine their relative magnitude in explaining the links of romantic conflict to problem gambling. We hypothesized that the serial mediation pathway will be significant, in which the link between romantic conflict to gambling is partially explained by negative affect to coping motives.

## 2. Materials and methods

### 2.1. Participants

A total of *N* = 206 partnered gamblers were recruited via Qualtrics Panels. To be included in the study, participants had to be regular gamblers (i.e., report gambling at least once a week), be 19 years of age or older (legal drinking and gambling age in the jurisdiction in which REB approval was obtained), currently be in Canada or the United States, be currently in a romantic relationship of at least three months’ duration and in which they reported seeing their partner in person at least three times per week. These last two inclusion criteria were used to help recruit only couples in more serious relationships and with a sufficient frequency of in-person contact to allow for in-person conflict behaviors, as there is evidence of different conflict styles and consequences in long-distance relationships (Lee et al., 2016). Recruited participants had a mean age of 44.72 years (*SD* = 15.26), with an age range of 19–78 years. The sample consisted of a majority of men (64%, *n* = 132).

A majority identified as White (80.6%; *n* = 166). A total of *n* = 19 identified as Arab, South Asian, or East Asian (9.2%). Eleven (5.3%) individuals identified as Black. Four (1.9%) identified as Latin American and *n* = 3 (1.4) identified as Indigenous. Six individuals identified multiple ethnicities (White and one other, counted in the non-White category endorsed) and *n* = 3 (1.5%) identified as other/prefer not to answer regarding ethnicity.

Most of the sample reported being in mixed-gender relationships, *n* = 193 (93%), and reported living with their current romantic partner at the time of study, *n* = 181 (88%). Over half the sample (*n* = 123, 59.7%) reported being married to their current partner, and the remaining participants were mostly engaged (*n* = 11, 5.3%), common law (*n* = 15, 7.3%), or exclusively dating (*n* = 42, 20.4%), with a minority reporting casually dating (*n* = 15, 7.3%). The mean duration of the participants’ current relationship was 15.29 years, *SD* = 13.83.

### 2.2. Measures

The following scales were administered to all participants as part of a larger study and used in the presented analyses. See Table 1 for descriptive statistics, internal consistencies, and bivariate correlations. Romantic conflict, negative affect, and problem gambling were all measured by querying the prior seven days, whereas the original validated versions query discrepant intervals of time (e.g., 12 months vs. 30 days vs. 7 days). Both versions (original versions and seven-day versions) of the negative affect and problem gambling measures were included in the battery for the present study, though the seven-day versions were used for analyses for consistency across measures. The original versions were included to validate the seven-day versions. A seven-day version of romantic conflict has already been validated (Lambe et al., 2015), and coping motives was not assessed in a specific timeframe, as is typical for motives assessment (e.g., Cooper, 1994).

**TABLE 1:** Correlations and descriptive statistics.

	1	2	3	4	5	6	7	8	9	10
1. Conflict^a^	–									
2. Negative affect^b^	0.667***	–								
3. Depression^b^	0.633***	0.963***	–							
4. Anxiety^b^	0.674***	0.973***	0.902***	–						
5. Stress^b^	0.657***	0.974***	0.902***	0.933***	–					
6. Coping motives^c^	0.455**	0.524***	0.536***	0.559***	0.517***	–				
7. Gambling problems^d^	0.636***	0.757***	0.728***	0.784***	0.718***	0.716***	–			
8. Age	−0.319***	−0.431***	−0.383***	−0.431***	−0.408***	−0.461***	−0.437***	–		
9. Relationship length	−0.236***	−0.404***	−0.402***	−0.400***	−0.380***	−0.375***	−0.386***	0.711***	–	
10. Gender^e^	−0.162*	−0.226**	−0.200**	−0.242***	−0.199**	−0.161*	−0.272***	0.082	0.060	–
Range (in sample)	7–63	0–126	0–42	0–42	0–42	5–20	0–24	19–78	3–642	
Mean (*SD*)	22.40 (15.20)	39.34 (38.11)	13.27 (13.53)	12.91 (13.33)	13.86 (12.89)	11.61 (4.48)	6.77 (8.05)	44.72 (15.26)	183.5 (166.00)	64% men
Internal consistency	0.95	0.98	0.96	0.95	0.95	0.89	0.97	–	–	–
Clinical cut-off	–	–	10–13–mild 14–20—moderate 21+—severe^b^	8–9—mild 10–14—moderate 15+—severe^b^	15–18—mild 19–25—moderate 26+—severe^b^	*M* = 11 in a sample of problem gamblers^d^	3–7—moderate 8+—problem^d^	–	–	–

^a^Partner-Specific Rejecting Behaviors Scale (Murray et al., 2003).

^b^Negative affect measured by combining the subscales from the Depression, Anxiety, and Stress Scale (DASS-21; Lovibond and Lovibond, 1995).

^c^Gambling Motives Questionnaire, coping motives subscale (Stewart and Zack, 2008).

^d^Problem Gambling Severity Index (PGSI; Ferris and Wynne, 2001).

^e^Negative correlation indicates higher levels in men; positive correlation indicates higher levels in women.

*p < 0.05, **p < 0.01, ***p < 0.001.

#### 2.2.1. Partner-specific rejecting behaviors scale

Romantic conflict was assessed using the 7-item Partner-Specific Rejecting Behaviors Scale (Murray et al., 2003). Participants were asked to report on their rejecting, critical, or dismissive behavior towards their partner in the last week (e.g., “I was angry or irritated with my partner”) on a Likert scale from *1-strongly disagree* to *9-strongly agree*. Items were summed, with higher scores (out of 63) indicating more severe conflict behavior. The seven-day timeframe was validated [e.g., convergent validity with the original scale) in Lambe et al. (2015)]. This scale has shown strong psychometric properties, including excellent internal consistency (α = 0.93; Mackinnon et al., 2012), which was supported in our sample, α = 0.95.

#### 2.2.2. Depression, anxiety, and stress scale

Negative affect was measured using the short form Depression, Anxiety, and Stress Scale (DASS-21; Lovibond and Lovibond, 1995). This scale is comprised of 21 items that ask participants to rate how much each statement (e.g., “*I felt I had nothing to look forward to*,” “*I found myself getting agitated*”) applied to them over the specified timeframe on a 4-point Likert scale from *0*-*did not apply to me at all* to *3-applied to me very much, or most of the time.* The scale is comprised of three subscales, assessing depression, anxiety, and stress. The subscales have strong psychometric properties, α = 0.94 for depression, α = 0.87 for anxiety, α = 0.91 for stress, and demonstrated factor structure and concurrent validity (Antony et al., 1998). As per author recommended scoring, scores were summed and doubled to be comparable to the original 42 item measure, producing a range from 0 to 126 (see Table 1 for cut scores and sample means). The seven-day version of the combined scale significantly correlated with the original 30-day version in our sample, *r* = 0.95, *p* < 0.001, and had excellent internal consistency, α = 0.98. Subscales, used in supplemental analyses, also significantly correlated with the original 30-day versions, as follows: depression *r* = 0.92, anxiety *r* = 0.93, stress *r* = 0.90, *p*s < 0.001. The subscales also had excellent internal consistency, αs > 0.95.

#### 2.2.3. Gambling motives questionnaire-revised

Gambling to cope was measured using the coping subscale of the four factor Gambling Motives Questionnaire (Schellenberg et al., 2016)—an adapted version of the 15-item GMQ (Stewart and Zack, 2008) which itself was originally adapted from the Drinking Motives Questionnaire (Cooper et al., 1992). This scale includes four gambling motives: coping, enhancement, social, and financial motives. Only the coping gambling motives subscale was utilized in the present study due to the theorized link between conflict and gambling problems. The coping gambling motives subscale has five items assessing how often participants gamble for each of the reasons given, rated on 4-point Likert scales from *1-almost never/never* to *4-almost always/always*, yielding a sum score from 5 to 20. Coping items include “*to forget your worries*” and “*to cheer you up when you’re in a bad mood*.” The GMQ-R shows a strong factor structure and good psychometric properties in samples of gamblers and problem gamblers, including an acceptable internal consistency of the coping motives subscale (α = 0.71; Schellenberg et al., 2016). The coping subscale showed good internal consistency in our sample, α = 0.89.

#### 2.2.4. Problem gambling severity index

Problem gambling severity was assessed using the Problem Gambling Severity Index (PGSI), a nine-item self-report measure assessing various gambling problems, such as betting more than one could afford, experiencing tolerance, and guilt over one’s gambling behavior. Items are rated on a 4-point Likert scale from *0-Never* to *3-Almost always.* Scores from the nine items were summed for a total score, which produced a possible range from 0 to 27, where higher scores indicated more problem gambling. The PGSI assessing problem gambling over the last year shows good internal reliability, α = 0.84, and strong sensitivity and specificity in detecting DSM-IV pathological gambling diagnoses—a precursor to DSM-5’s gambling disorder diagnosis (Ferris and Wynne, 2001; American Psychiatric Association [APA], 2013). Additionally, the seven-day version of the PGSI significantly correlated with the original 12-month version in our sample, *r* = 0.96, *p* < 0.001, and had excellent internal consistency, α = 0.97.

### 2.3. Procedure

Qualtrics Panels, a survey management service, was used to recruit and gather the current data from a large pool of potential participants. A total of 206 participants were recruited in July 2021 and deemed eligible for the larger study based on our inclusion and exclusion criteria. An additional 623 individuals attempted to participate in the study but were excluded for a variety of reasons: not being in a relationship (*n* = 185); not gambling at least once per week in the last year (*n* = 182); not being 19 years of age or older (*n* = 147); failing validity checks (such as highly improbable responses, highly improbable survey completion time, *n* = 35); duplicate entries (*n* = 33); not being in Canada or the United States (*n* = 24); relationship shorter than three months (*n* = 11); and seeing their romantic partner face-to-face fewer than three times per week (*n* = 6). Eligible participants who provided informed consent and were subsequently administered a brief computerized battery of questionnaires including, but not limited to those employed in the present study (total duration of thirty minutes). The study was approved by the Dalhousie University Health Sciences Research Ethics Board (approval #: 2020-5368).

All analyses were conducted using IBM SPSS Statistics 27. The serial multiple mediation model was tested using PROCESS, a regression-based tool that tests for both direct and indirect effects (Hayes, 2014). The mediation model was run using PROCESS Model 6 to test the impact of negative affect and, in turn, coping motives as serial mediators of the association between romantic conflict and gambling problems. Additionally, age and relationship length were added as covariates to the model due to their significant correlations with most variables in the serial mediation model and the substantial variability in relationship length and age in our sample. All indirect effects were run with follow-up bootstrap analyses with 5,000 resamples from which 95% bias-corrected and accelerated confidence intervals (CIs) were estimated.

## 3. Results

Bivariate correlations between variables of interest can be found in Table 1. All variables were significantly correlated with each other, with correlations ranging from *r* = 0.46 to 0.97 in magnitude, indicating moderate to large effect sizes. On average, the current sample reported: mild to moderate depressive affect, moderate levels of anxious affect, below the mild cut-off of stress, coping motives consistent with a problem gambling sample, and moderate gambling problems in comparison to established norms or clinical cut-offs on these scales (Table 1).

While the authors originally intended to examine the DASS-21 subscales individually within the same model, the data revealed that the subscales overlapped too highly to permit this. Pairwise correlations and variance inflation factor (VIF) values were calculated to assess for multicollinearity. Correlation coefficients between the DASS-21subscales ranged from 0.90 to 0.93 (Table 1). VIF values ranged from 6.1 to 9.3. While there exists no single consensus cut-off value to avoid problems with multicollinearity, most experts suggest cut-offs of *r*s > 0.80 and VIF > 5 (Vatcheva and Lee, 2016). These values suggest that multicollinearity would be an issue if the DASS-21subscales were used as separate variables in a multiple-mediators model. Thus, we combined the three DASS-21subscales into a single score, measuring general negative affect. While a three-factor model was shown to be a better fit than a single-factor model in the original psychometric validation of this scale, the current sample’s responses yielded meaningfully higher correlations between the subscales compared to the original data, which ranged from *r*s = 0.54 to 0.65, suggesting a less meaningful distinction between these negative affective states in this sample of regular gamblers (Lovibond and Lovibond, 1995). Additionally, some studies have found comparable clinical utility (i.e., sensitivity and specificity for mood and anxiety disorders) of the combined scale to the subscales (e.g., Dahm et al., 2013). Additional sensitivity analyses were conducted with each DASS-21 subscale separately (Table 2).

**TABLE 2:** Comparison of the indirect effects of conflict on gambling problems through depression, anxiety, and stress subscales of the DASS-21 and coping motives and the specific indirect effects.

Effects	β	SE	LL	UL
**Depression**				
Total indirect*	0.34	0.05	0.24	0.45
(1) Conflict → depression → problem gambling*	0.22	0.04	0.14	0.31
(2) Conflict → coping motives → problem gambling	0.05	0.03	−0.01	0.12
(3) Conflict → depression → coping motives → problem gambling*	0.07	0.02	0.03	0.11
**Contrasts**				
Model 1 vs. model 2*	0.17	0.06	0.06	0.28
Model 1 vs. model 3*	0.15	0.05	0.06	0.24
Model 2 vs. model 3	−0.02	0.04	−0.10	0.07
**Anxiety**				
Total indirect*	0.39	0.06	0.28	0.51
(1) Conflict → anxiety → problem gambling*	0.27	0.05	0.18	0.39
(2) Conflict → coping motives → problem gambling	0.04	0.04	−0.02	0.12
(3) Conflict → anxiety → coping motives → problem gambling*	0.07	0.02	0.03	0.12
**Contrasts**				
Model 1 vs. model 2*	0.23	0.07	0.10	0.37
Model 1 vs. model 3*	0.20	0.06	0.10	0.32
Model 2 vs. model 3	−0.03	0.05	−0.13	0.08
**Stress**				
Total indirect*	0.32	0.05	0.21	0.42
(1) Conflict → stress → problem gambling*	0.21	0.05	0.12	0.30
(2) Conflict → coping motives → problem gambling	0.05	0.04	−0.01	0.13
(3) Conflict → stress → coping motives → problem gambling*	0.06	0.02	0.02	0.10
**Contrasts**				
Model 1 vs. model 2*	0.16	0.06	0.04	0.27
Model 1 vs. model 3*	0.15	0.05	0.06	0.25
Model 2 vs. model 3	−0.01	0.05	−0.10	0.10

Indirect effects where the LL (lower limit) and UL (upper limit) do not cross zero are considered significant indirect effects and are indicated with an asterisk (*). Model comparisons where the 95% CI LL and 95% CI UL do not cross zero are considered significant contrasts and are indicated with an asterisk (*). Analyses were run with age, relationship length, and gender as covariates.

Age, relationship length, and gender were included in the serial mediation model as covariates due to their significant correlations with several variables included in the serial mediation model. Despite age and relationship length being strongly correlated with each other (Table 1), these variables were found to have a VIF = 2.02, suggesting low risk of multicollinearity by including both in the same model.

**FIGURE 1:**
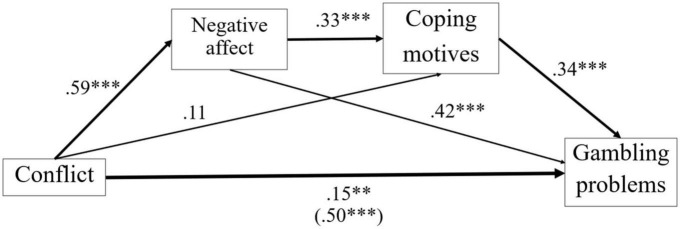
Serial mediaton model. Path values indicate standardized coefficients (β). *p* < 0.01^**^, *p* < 0.001^***^.

The serial mediation model was run using the combined negative affect scores and coping gambling motives as mediators between romantic conflict and problem gambling. Regarding direct effects observed in Figure 1, conflict had a significant and positive effect on negative affect, a_1_, β = 0.59, *p* < 0.001, and non-significant effect on coping motives, a_2_, β = 0.11, *p* = 0.20. Negative affect had a positive effect on coping gambling motives when the effects of conflict were controlled, d_21_, β = 0.33, *p* < 0.001, as did negative affect on gambling problems when conflict was controlled, b_1_, β = 0.42, *p* < 0.001, and as did coping gambling motives on gambling problems when conflict and negative affect were controlled, b_2_, β = 0.34, *p* < 0.001. The total effect of conflict on gambling problems was significant, c, β = 0.50, *p* < 0.001. Additionally, the direct effect of conflict on gambling problems remained significant when negative affect and coping motives were controlled, c’, β = 0.15, *p* = 0.008, although its magnitude was much reduced.

Relationship length significantly and negatively covaried with negative affect, β = −0.22, *p* = 0.004, but not with coping motives, β = 0.05, *p* = 0.58, gambling problems, β = 0.01, *p* = 0.87, or the total effect, β = −0.09, *p* = 0.25. Age significantly and negatively covaried with both coping motives, β = −0.36, *p* < 0.001, and the total effect, β = −0.23, *p* = 0.004. However, age did not significantly covary with negative affect, β = −0.07, *p* = 0.40 or with gambling problems, β = −0.07, *p* = 0.25. Gender did not significantly covary with negative affect, β = −0.08, *p* = 0.14, or coping motives, β = −0.06, *p* = 0.37. Gender covaried with both gambling problems, β = −0.13, *p* = 0.003, wherein identifying as a man was associated with more gambling problems, and with the total effect, β = −0.19, *p* < 0.001, wherein identifying as a man was associated with a significantly stronger *c* path in the model.

Mediation effects were also observed (Table 3). As hypothesized, the total indirect effect of romantic conflict on gambling problems via negative affect and coping motives was significant, a_1_*b_1_ + a_1_*d_21_*b_2_ + a_2_*b_2_, β = 0.35, 95% CI [0.24, 0.46]. Additionally, two of the three component mediator indirect effects were significant. The fully standardized indirect effect between conflict and gambling problems through negative affect was positive and significant, a_1_*b_1_, β = 0.25, 95% CI [0.16, 0.35], although the single mediator indirect effect through coping gambling motives was not significant, a_2_*b_2_, β = 0.04, 95% CI [−0.02, 0.11]). Additionally, the fully standardized serial indirect effect from conflict to gambling problems via negative affect and, in turn, coping motives was positive and significant, a_1_*d_21_*b_2_, β = 0.07, [0.03, 0.11]. These standardized coefficients represent amount of explained variance in gambling problems per standard deviation increase in conflict via the mediating variable. For example, with the total mediation effect, for every SD increase in conflict, gambling problems increased by 0.35 SD units via negative affect and coping motives. In other words, per SD increase in romantic conflict, gambling problems increase by 0.50 SD units; however, this is reduced to 0.15 SD units after controlling for both negative affect and coping motives.

**TABLE 3:** Comparison of the indirect effects of conflict on gambling problems through negative affect and coping motives and the specific indirect effects.

Effects	β	SE	LL	UL
Total indirect*	0.35	0.06	0.24	0.47
(1) Conflict → negative affect → problem gambling*	0.24	0.05	0.16	0.35
(2) Conflict → coping motives → problem gambling	0.04	0.03	−0.02	0.11
(3) Conflict → negative affect → coping motives → problem gambling*	0.07	0.02	0.03	0.11
**Contrasts**				
Model 1 vs. model 2*	0.21	0.06	0.09	0.34
Model 1 vs. model 3*	0.18	0.05	0.09	0.30
Model 2 vs. model 3	−0.03	0.04	−0.11	0.06

Indirect effects where the LL (lower limit) and UL (upper limit) do not cross zero are considered significant indirect effects and are indicated with an asterisk (*). Model comparisons where the 95% CI LL and 95% CI UL do not cross zero are considered significant contrasts and are indicated with an asterisk (*). Analyses were run with age, relationship length, and gender as covariates.

Contrasts were run to compare the relative magnitude of each indirect effect (Table 3). The single mediation between conflict and gambling problems via negative affect was significantly larger than the other two indirect effects (single mediation via coping motives and serial mediation through both mediators). The single mediation via coping motives and serial mediation effects were not significantly different from one another. Overall, the model significantly predicted 52% of the variance in gambling problems, *F*(1, 173) = 46.69, *R* = 0.72, *R^2^* = 0.52, *p* < 0.001.

The above model was also re-run three times with each of depressive affect, anxious affect, and stress (DASS-21subscales) in place of the combined negative affect sum score (Table 2) to examine whether one or more of the specific forms of negative affect were carrying the mediational findings. The results were virtually unchanged for each model with the same significant paths, mediational findings, and significant contrasts (relative magnitude of mediational paths) as reported for the main model using general negative affect as the first mediator. Additionally, the main analyses were re-run without the covariates of age, relationship length, and gender, and the results were not meaningfully different (see Supplementary Table 1).

## 4. Discussion

The current study provided evidence for one possible explanation for the link between romantic conflict and gambling problems, namely serial mediation via negative affect and, in turn, coping gambling motives. As hypothesized, indirect effects via negative affect (single mediation) were significant in predicting gambling problems; however, indirect effects via coping gambling motives (single mediation) were non-significant in predicting gambling problems while controlling for other variables in the serial mediation model. Additionally, the model indicated that a direct effect between conflict and gambling problems remained after controlling for negative affect and coping gambling motives, suggesting the presence of other mediators not accounted for in the current model. Overall, the model explained almost half of the variance in problem gambling in our sample. The present study continues to highlight the interpersonal context in which gambling problems are embedded (Kalischuk et al., 2006; Cowlishaw et al., 2016).

Consistent with our primary hypothesis, the significant chained mediational pathway suggests regular gamblers may respond to negative affect following romantic conflict by gambling to cope which in turn puts them at risk for gambling problems. Our findings are consistent with research that shows strong associations between conflict and gambling problems (Dowling et al., 2016), that conflict behaviors robustly predict negative affect (Liu and Chen, 2006; El-Sheikh et al., 2013), and that coping gambling motives are an important predictor of gambling problems (Stewart and Zack, 2008). However, previous literature has suggested a stronger link between depressive affect and gambling than between anxious affect and gambling (Barrault et al., 2019), while comparably strong links between different forms of negative affect (i.e., depression, anxiety, and stress) were supported by additional analyses with the present data. Indeed, the current data suggests that the specific type of negative affect makes little difference in terms of its strength of association to gambling problems. Despite the clear bidirectional link between relationship functioning and mental health, meta-analysis suggests the stronger effect is actually relationship functioning predicting mental health variables (Braithwaite and Holt-Lunstad, 2017), the direction tested in and supported by our study. Our study also adds to the extant coping gamblingmotives literature by expanding our understanding of what individuals who gamble to cope may be responding to, specifically by testing the associations between romantic conflict and gambling to cope via resultant negative affect. This study also extends the findings from Lambe et al.’s (2015) study, which established mediation from romantic conflict to alcohol problems via coping drinking motives, in important ways. Primarily, this study extends our understanding from drinking motives and alcohol problems to gambling motives and problem gambling. The current study also used a sample of adults representing a broad age range drawn from the general population rather than a narrower college-aged sample. Lastly, the present analyses included and directly tested negative affect, an assumed mediator in Lambe et al. (2015). The inclusion of negative affect strengthens the theoretical construct of coping motives (that individuals engage in gambling to cope with distressing affective states) by directly testing for negative affective states as a mediator between a stressor (romantic conflict) and coping motives.

Coping gambling motives was not a significant single mediator between romantic conflict and gambling problems in the serial mediation model, despite significant serial mediation. The non-significant pathway via coping gambling motives suggests that coping motives requires the presence of negative affect to help explain the link from conflict to gambling problems. It appears that coping motives only contributes to gambling problems associated with romantic conflict insofar as coping motives are predicted by negative affect. This also suggests that negative affect sufficiently mapped onto the measure of coping motives to explain the variance in coping motives. On the other hand, negative affect appeared as a significant single mediator even when accounting for the effects of coping motives. Notably, the coping motives measure remained a non-significant mediator in each of the models with the DASS-21 subscales separately (see sensitivity analyses). This suggests that the measure of coping motives was unable to explain much of the variance in negative affect predicting problem gambling. It is possible that a coping motives measure that includes items tapping into coping-with-stress in addition to coping-with-depression and coping-with-anxiety, an extension of the factor structure found in the alcohol motives literature (Grant et al., 2007), may better capture the range of negative affect associated with romantic conflict. It may also be that a measure of negative affect that could more effectively distinguish between different negative affective states may better map onto coping motives sub-scales (e.g., coping-with-depression), with overall greater specificity between measures. This may reduce the variance explained by negative affect alone without coping gambling motives.

Another possibility is that negative affect is associated with gambling problems via another serial mediator, such as enhancement motives. Despite less overall evidence and a less clear theoretical link, the alcohol literature has indeed demonstrated an association between negative affect and drinking frequency in those high in enhancement motives using daily diary methodology (Armeli et al., 2010). Additionally, some research has found that negative urgency (impulsive behavior in response to negative mood states) is associated with drinking behavior via enhancement motives (Anthenien et al., 2017). Subtyping research on gambling motives has also suggested that coping-based gamblers tend to gamble mostly for negative reinforcement (i.e., coping) but in conjunction with enhancement-motivated gambling, distinct from a subtype of gamblers who primarily gamble for enhancement purposes (Stewart et al., 2008). Lastly, gamblers may respond to negative affect following conflict in other ways to attempt to cope that lead to further gambling problems that are not captured by the coping motives scale of the GMQ (Stewart and Zack, 2008). For instance, individuals may gamble as a strategy to avoid their partner or further conflict. Additionally, some problem gamblers have reported gambling as a source of meaning-making or to feel “normal”; feeling negative mood states following conflict may drive individuals to gamble to feel “normal” (Yakovenko et al., 2016).

Overall, a significant direct effect between conflict and problem gambling remained after accounting for the mediated pathways through depressive affect and coping motives, suggesting other mediating mechanisms besides those tested in the current model. One possibility is the role of anger, which was not included in the present model or captured in our measure of negative affect. Prior research has evidenced clinically significant anger problems in many problem gamblers (i.e., 65%), as well as a strong association between perpetration of intimate partner violence (an extreme form of relationship conflict) and anger in problem gamblers (Korman et al., 2005, 2008; Dowling et al., 2016). Additionally, in a large sample of individuals seeking treatment for substance use disorders, those with problem gambling reported significantly more anger problems than their non-problem gambling treatment-seeking counterparts (Collins et al., 2005). Another study similarly found that substance abusers who endorsed violent tendencies were three times more likely to be problem gamblers (Cunningham-Williams et al., 2007). These findings suggest that problem gamblers may experience angry affect to a greater degree than other populations, including those who abuse substances, and thus that anger and gambling to cope with anger may play unique roles in the link between romantic conflict to gambling problems that may not be captured by the negative affect measure used in this study (i.e., the DASS-21) or by coping motives measures adapted from alcohol motives. This suggests the need for the development and validation of a measure of coping with anger as a motivation for gambling, and for the inclusion of measures of anger in future studies of the links of romantic conflict to problem gambling.

Another possible mediator between conflict and gambling problems are financial motives. Indeed, financial problems are associated with increased family conflict for gamblers (Carr et al., 2018). Moreover, conflict about money was among the most common negative impacts reported by family members of a problem gambler (Dowling et al., 2016), highlighting one possible contributing factor to the high rates of conflict reported among couples where one is a problem gambler. Although not yet directly tested, it is possible that individuals have heightened motivation to gamble for financial reasons or to chase losses (Blaszczynski and Nower, 2006) following conflict about finances as a form of “problem solving” (albeit maladaptive problem solving). This would be consistent with the association between deprivation and gambling problems (Lloyd et al., 2021).

Regarding covariates, relationship length negatively predicted all negative affect variables, which is consistent with the widely reported protective effects of marriage (Uecker, 2012). Inconsistent with the original validation of the Gambling Motives Questionnaire (Stewart and Zack, 2008), our model indicated a significant negative relationship between age and coping motives; this may be due to the present study having recruited an older sample on average. Further research would be required to probe whether this finding replicates and the possible mechanism underlying this association. Additionally, the total effect path between conflict and gambling problems was negatively predicted by age, suggesting that romantic conflict may be a more significant problem gambling risk factor for younger vs. older adults. Gender also significantly predicted gambling problems, consistent with epidemiology indicating more gambling problems in men than women (Hing et al., 2016). Additionally, gender predicted the total effect, suggesting that conflict may be particularly risky for exacerbating gambling problems for men. The total effect size was not meaningfully different in analyses not controlling for gender (see Supplemental materials). Notably, gender did not significantly predict negative affect, despite consistent evidence of women typically reporting more negative affect and disorders associated with negative affect than men (Leach et al., 2008). However, as some studies report equivalent rates of depressive and anxiety disorders by gender in problem gamblers, this may explain why women in this sample did not report higher levels of depression, anxiety, and stress (Yakovenko and Hodgins, 2018).

The present study findings are limited by cross-sectional design and thus cannot be used to draw causal interpretations. Some have argued that mediation models should not be tested with cross-sectional data (Maxwell et al., 2011). However, other statisticians have argued that cross-sectional mediation is entirely acceptable, as long as it is interpreted accurately based on the inherent limitations, and can play an important role in providing a preliminary foundation for a novel theoretical mediation model to then justify longitudinal research to corroborate the putative causal chain (Hayes and Rockwood, 2020). Additionally, our hypothesized model was informed by strong empirical evidence on the temporal effects of relationship functioning on various forms of psychopathology (Braithwaite and Holt-Lunstad, 2017). Future research is necessary to confirm these findings by incorporating longitudinal designs; specifically, four waves of cross-lagged panel analysis could demonstrate if these pathways are contributing to exacerbated problem gambling over time (e.g., Mushquash et al., 2013). Additionally, ecological momentary assessment would provide rich data into the event-level mechanisms (Votaw and Witkiewitz, 2021), i.e., if individuals experience negative affect after specific romantic conflict events and endorse gambling to cope later the same day or on the day following a romantic conflict. Additionally, this cross-sectional data was collected following the COVID-19 pandemic, making it difficult to draw conclusions about how these results may compare to pre-pandemic gambling problems. While evidence is mixed (Hodgins and Stevens, 2021), the most reliable finding is that those with pre-pandemic gambling problems were more likely than others to increase their gambling behavior or experience worsened problems associated with gambling with the onset of the pandemic (Brodeur et al., 2021). Additionally, reports of intimate partner violence roughly doubled in both frequency and severity during the pandemic (Gosangi et al., 2021). However, how the link between these variables may have changed during the pandemic is unclear.

While the present study only included the gambler’s perception of romantic conflict, conflict is often conceptualized as a dynamic and dyadic variable (e.g., Mackinnon et al., 2012; Lambe et al., 2015). Additionally, these data only include measures of self-reported conflict behaviors enacted by the participants onto their partners, e.g., yelling at their partner. However, problem gamblers report both higher conflict perpetration and conflict victimization compared to the general population (Dowling et al., 2016). Furthermore, these reports tend to go together—three quarters of problem gamblers who report any intimate partner violence report bidirectional violence (Suomi et al., 2019). Some studies have suggested that victimization acts as an antecedent to gambling problems, which may involve coping motives not captured by the GMQ, such as gambling to escape a violent home situation or to cope with the traumatic experience (Afifi et al., 2010; Echeburúa et al., 2011; Dowling et al., 2016). Future studies may wish to include both partners’ perceptions of conflict and/or contributions to the conflict (i.e., conflict enacted toward and received from the partner; see Basso et al., 2023(Basso et al., 2023), to get a fuller understanding of the impact of conflict on gambling problems). Future studies may also want to examine gender moderated mediation, as prior work with conflict, coping motives, and alcohol problems has demonstrated a stronger coping mediation pathway in women than men (Lambe et al., 2015). As with all self-reported behavior, but particularly for self-reports of undesirable behaviors such as enacting conflict toward one’s partner, response bias may interfere with the validity of the measure (Mathie and Wakeling, 2011). However, much research has reliably demonstrated hypothesized findings from self-reported conflict behavior, and that one’s own perception of conflict may be the stronger predictor of addictive behaviors (Derrick et al., 2009); additionally, respondent participation was anonymous, which may reduce response bias.

We used a measure of negative affect as a mediator. Nonetheless, it is possible that regular gamblers gamble to cope with symptoms beyond affect. Indeed, another study by Keough et al. (2018) used a global measure of depressive symptoms that went beyond depressed affect (e.g., insomnia, anhedonia, cognitive symptoms) and found mediation of the link of another interpersonal difficulty (attachment issues) to gambling to cope. Future studies of the conflict to problem gambling link should include measures of emotional disorder symptoms that go beyond negative affect.

These data have important clinical implications. These findings further underscore the need to screen for conflict behavior in those seeking treatment for problem gambling – not merely due to the high prevalence rate in this population, but also due to the possible role of conflict in maintaining problem gambling observed here. Clinicians may also wish to target both negative affect and coping gambling motives in both individual treatment with a partnered gambler and in couples-based treatments for gamblers experiencing romantic conflict (Nilsson et al., 2019). Additionally, the significant direct effect of conflict on problem gambling after controlling for negative affect and coping motives suggests the importance of directly targeting conflict behavior as well (e.g., through communications training or perspective taking interventions (Rodriguez et al., 2021)). This is further supported by evidence that improving relationships has a stronger effect on improving mental health than the other way around (Braithwaite and Holt-Lunstad, 2017). These findings additionally have implications for relapse prevention. Indeed, problem gamblers cite experiencing negative emotions like depressive affect as a primary antecedent to relapse (11% of relapses; Hodgins and El-Guebaly, 2004). Our findings suggest that management of negative emotions following romantic conflict may be an important relapse prevention skill in mitigating relapse to problem gambling.

## Data availability statement

The raw data supporting the conclusions of this article will be made available by the authors, without undue reservation.

## Ethics statement

The studies involving human participants were reviewed and approved by the Dalhousie Research Ethics Board. The patients/participants provided their written informed consent to participate in this study.

## Author contributions

SHS, LR, SBS, IY, and RN-A secured the funding for the study with SHS leading the grant submission to Gambling Awareness Nova Scotia. SHS led the ethics application and the contract with Qualtrics Panel Surveys. AH organized the database, performed the statistical analyses, wrote the first draft of the manuscript, and made revisions, including conducting additional analyses, through the review process. All authors contributed to conception and design of this study, manuscript revision, read, and approved the submitted version.
