# Corrigendum: What explains the link between romantic conflict with gambling problems? Testing a serial mediational model

**DOI:** 10.3389/fpsyg.2023.1305966

**Published:** 2023-11-03

**Authors:** Amanda E. F. Hagen, Raquel Nogueira-Arjona, Simon B. Sherry, Lindsey M. Rodriguez, Igor Yakovenko, Sherry H. Stewart

**Affiliations:** ^1^Department of Psychology & Neuroscience, Dalhousie University, Halifax, NS, Canada; ^2^School of Psychology, University of Sussex, Falmer, United Kingdom; ^3^Department of Psychology, University of South Florida St. Petersburg Campus, Tampa, FL, United States; ^4^Department of Psychiatry, Dalhousie University, Halifax, NS, Canada

**Keywords:** gambling, problem gambling, romantic conflict, coping motives, negative affect

In the published article, there was an error. In the Abstract, there were errors in the reporting of beta values and corresponding confidence intervals. The Results section of the abstract previously stated, “Results: Results supported our hypothesis that the association between romantic conflict and gambling-related problems would be sequentially mediated through negative affect and coping gambling motives, β = 0.38, 95% CI [0.27, 0.39], and also showed a strong single mediation pathway through negative affect alone, β = 0.27, 95% CI [0.17, 0.38].”

The corrected section appears below:

“**Results**

Results supported our hypothesis that the model would explain a significant amount of variance in gambling-related problems, β = 0.35, 95% CI [0.24, 0.47], and that the association between romantic conflict and gambling-related problems would be sequentially mediated through negative affect and coping gambling motives, β = 0.07, 95% CI [0.03, 0.11], and also showed a strong single mediation pathway through negative affect alone, β = 0.24, 95% CI [0.16, 0.35].”

The authors apologize for this error and state that this does not change the scientific conclusions of the article in any way. The original article has been updated.

