# Supplementary material for: What explains the link between romantic conflict with gambling problems? Testing a serial mediational model
Source: Front Psychol. 2023 Jul 12;14:1018098. doi: 10.3389/fpsyg.2023.1018098 (PMC10370473; doi:10.3389/fpsyg.2023.1018098)
Supplement: Supplementary file 1 [file Table_1.pdf]

Supplemental Table 1. Comparison of the indirect effects of conflict on gambling problems through depression, anxiety, and stress subscales of the DASS-21 and coping motives and the specific indirect effects without covariates.

Effects	*β*	SE	LL	UL
Depression				
Total indirect*	.42	.05	.33	.52
1) Conflict → depression → problem gambling*	.24	.04	.15	.33
2) Conflict → coping motives → problem gambling*	.08	.03	.01	.15
3) Conflict → depression → coping motives → problem gambling*	.11	.02	.07	.16
Contrasts				
Model 1 vs model 2*	.16	.06	.03	.29
Model 1 vs model 3*	.13	.05	.03	.22
Model 2 vs model 3	-.03	.05	-.13	.07
Anxiety				
Total indirect*	.49	.05	.40	.60
1) Conflict → anxiety → problem gambling*	.32	.05	.23	.43
2) Conflict → coping motives → problem gambling	.05	.04	-.01	.13
3) Conflict → anxiety → coping motives → problem gambling*	.12	.02	.07	.18
Contrasts				
Model 1 vs model 2*	.27	.07	.13	.42
Model 1 vs model 3*	.20	.06	.10	.32
Model 2 vs model 3	-.07	.05	-.18	.04
Stress				
Total indirect*	.43	.05	.33	.53
1) Conflict → stress → problem gambling*	.25	.05	.16	.35
2) Conflict → coping motives → problem gambling	.07	.04	-.003	.15
3) Conflict → stress → coping motives → problem gambling*	.11	.03	.06	.17
Contrasts				
Model 1 vs model 2*	.18	.07	.04	.32
Model 1 vs model 3*	.13	.05	.03	.25
Model 2 vs model 3	-.04	.06	-.15	.07
Negative affect				
Total indirect*	.47	.05	.37	.58
1) Conflict → negative affect → problem gambling*	.30	.05	.20	.41
2) Conflict → coping motives → problem gambling	.04	.03	-.02	.13
3) Conflict → negative affect → coping motives → problem gambling*	.12	.03	.07	.17
Contrasts				
Model 1 vs model 2*	.24	.07	.11	.40
Model 1 vs model 3*	.18	.06	.07	.31
Model 2 vs model 3	-.07	.06	-.18	.04

Notes: Indirect effects where the LL (lower limit) and UL (upper limit) do not cross zero are considered significant indirect effects and are indicated with an asterisk (*). Model comparisons where the 95% CI LL and 95% CI UL do not cross zero are considered significant contrasts and are indicated with an asterisk (*). Analyses were run without covariates.
